# Effects of hydroxyl group in cyclo(Pro-Tyr)-like cyclic dipeptides on their anti-QS activity and self-assembly

**DOI:** 10.1016/j.isci.2023.107048

**Published:** 2023-06-07

**Authors:** Li Li, Zuxian Xu, Ruipin Cao, Jiaxin Li, Chang-Jer Wu, Yinglu Wang, Hu Zhu

**Affiliations:** 1 Fujian-Taiwan Science and Technology Cooperation Base of Biomedical Materials and Tissue Engineering, Engineering Research Center of Industrial Biocatalysis, Fujian Provincial Key Laboratory of Advanced Materials Oriented Chemical Engineering, Fujian Provincial Key Laboratory of Polymer Materials, College of Chemistry and Materials Science, Fujian Normal University, Fuzhou 350007, China; 2 Department of Food Science, National Taiwan Ocean University, Keelung 20224, Taiwan; 3 College of Chemical Engineering and Materials Science, Quanzhou Normal University, Quanzhou 362000, China

**Keywords:** Biomolecules, Microbiology, Bacteriology

## Abstract

We investigated the influence of hydroxyl groups on the anti-quorum-sensing (anti-QS) and anti-biofilm activity of structurally similar cyclic dipeptides, namely cyclo(*L*-Pro-*L*-Tyr), cyclo(*L*-Hyp-*L*-Tyr), and cyclo(*L*-Pro-*L*-Phe), against *Pseudomonas aeruginosa* PAO1. Cyclo(*L*-Pro-*L*-Phe), lacking hydroxyl groups, displayed higher virulence factor inhibition and cytotoxicity, but showed less inhibitory ability in biofilm formation. Cyclo(*L*-Pro-*L*-Tyr) and cyclo(*L*-Hyp-*L*-Tyr) suppressed genes in both the *las* and *rhl* systems, whereas cyclo(*L*-Pro-*L*-Phe) mainly downregulated *rhl*I and *pqs*R expression. These cyclic dipeptides interacted with the QS-related protein LasR, with similar binding efficiency to the autoinducer 3OC12-HSL, except for cyclo(*L*-Pro-*L*-Phe) which had lower affinity. In addition, the introduction of hydroxyl groups significantly improved the self-assembly ability of these peptides. Both cyclo(*L*-Pro-*L*-Tyr) and cyclo(*L*-Hyp-*L*-Tyr) formed assembly particles at the highest tested concentration. The findings revealed the structure-function relationship of this kind of cyclic dipeptides and provided basis for our follow-up research in the design and modification of anti-QS compounds.

## Highlights

• Anti-QS activity of cyclo(Pro-Tyr)-like peptides were systemically assayed
• How the OH group regulated the activity and cytotoxicity has been elucidated
• Absence of OH group altered the acting pathway on QS system
• Introduction of OH groups proved to significantly enhance self-assembly tendency

## Graphical abstract

**Figure:**
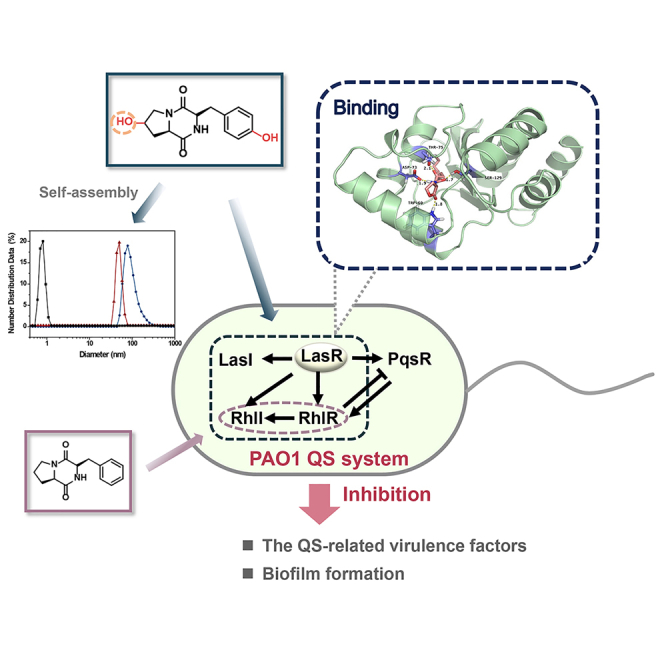

## Introduction

Quorum sensing (QS) is a communication system that relies on autoinducers (AIs) to regulate population behavior among bacteria.^1^ Once the concentration of AIs reaches a certain threshold, they will bind to the receptors and then activate or inhibit the transcription and expression of several target genes, thereby regulating the biological population of bacteria and bacterial pathogenicity,^2^ such as bioluminescence, biofilm formation, differentiation, extracellular polysaccharides production, motility, antibiotics production, and so on. Most antibiotics inhibit the synthesis of genetic material DNA/RNA or proteins involved in metabolic pathways in bacteria.^3^ However, bacteria can develop resistance to these mechanisms, leading to many antibiotics’ failure.^4^ In contrast, the quorum sensing inhibitors (QSIs) target the QS system and inhibit the pathogenic process of bacteria,^5^^,^^6^^,^^7^ which rarely affects the average growth of bacteria and dramatically reduces the probability of bacterial resistance. According to the Centers for Disease Control and Prevention (CDC) report on the threat of antibiotic resistance, multidrug-resistant (MDR) bacteria pose a serious threat to public health, with some types of MDR *Pseudomonas aeruginosa* resistant to almost all antibiotics, even carbapenems.^8^ *P. aeruginosa* is a common opportunistic human pathogen that can cause various healthcare-associated infections, including pneumonia, bloodstream infections, urinary tract infections, and surgical site infections, and is particularly deadly for patients with chronic lung disease.^9^^,^^10^^,^^11^^,^^12^^,^^13^ Numerous studies have shown that the expression of the pathogenic virulence factors of *P. aeruginosa* is regulated by its QS system,^14^^,^^15^^,^^16^ thus making *P. aeruginosa* a suitable model for studying QSIs against gram negative bacteria.

**Figure 1:**
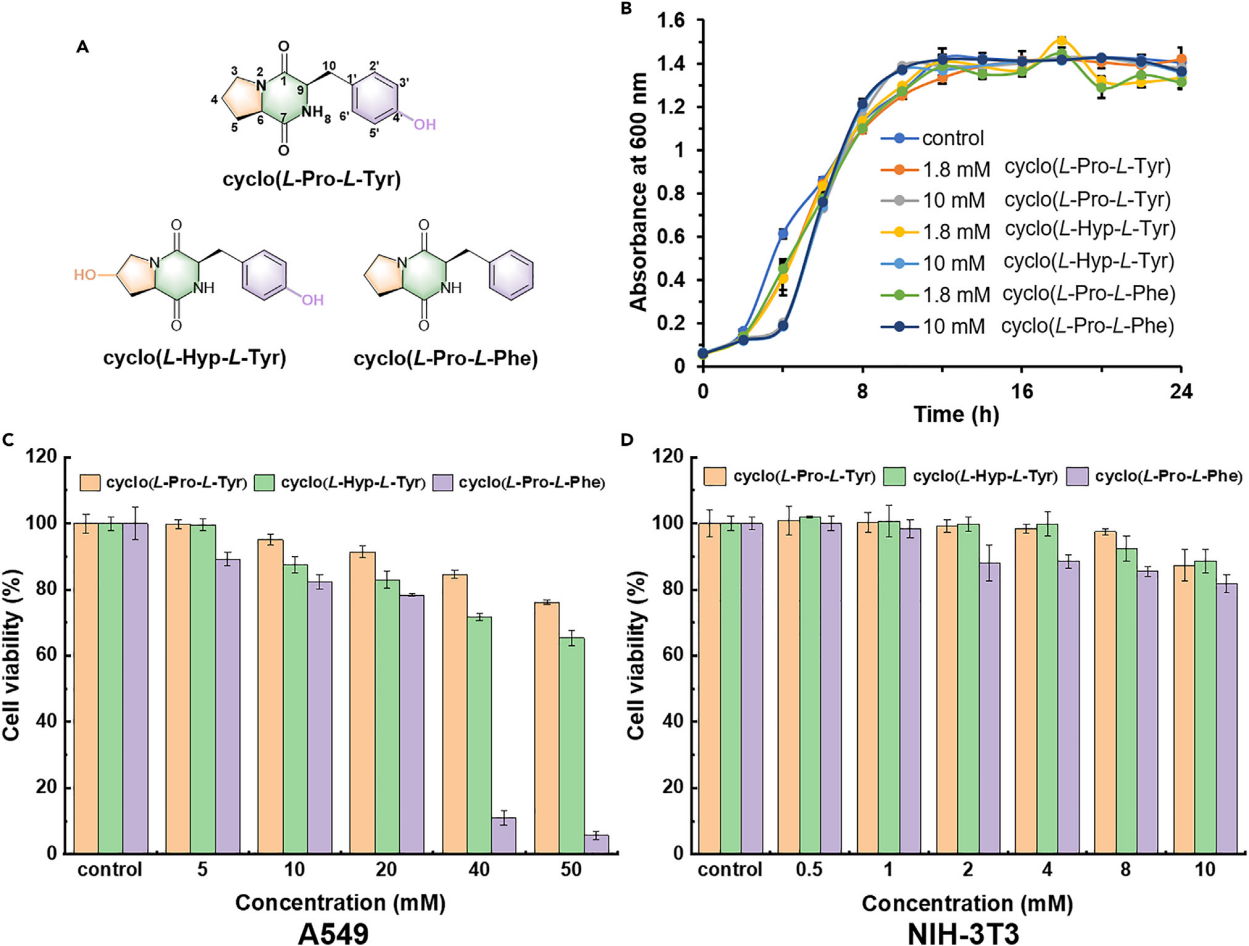
Chemical structure and biocompatibility of CDPs (A) Chemical structures of CDPs. (B) Growth curve of *P. aeruginosa* PAO1 treated with CDPs at concentrations of 1.8 mM and 10 mM, PBS was used as the blank control. Cytotoxicity of CDPs on (C) A549 cells and (D) NIH-3T3 cells. Values are presented as mean ± SD (n = 3).

Cyclic dipeptides (CDPs) are widespread in nature and exhibit various biological activities. For instance, cyclo(His-Pro) was discovered as an endogenous compound in mammals’ brains, and shows potential as a treatment for neurodegenerative diseases^17^^,^^18^; cyclo(*L*-Phe-*L*-Pro) and cyclo(*L*-Phe-*trans*-4-OH-*L*-Pro), both extracted from *Lactobacillus plantarum* (MiLAB 393) isolated from grass silage, have been proved to be antifungal^19^; cyclo(*L*-Pro-*D*-Arg) isolated from the *Bacillus cereus* showed antibacterial and antitumor activity^20^; cyclo(*L*-Leu-*L*-Tyr) isolated from sponge-associated fungi *Penicillium* sp.^21^ and cyclo(*L*-Leu-*L*-Pro) from *Bacillus amyoliquefaciens*^22^ were reported to inhibit biofilm formation of *Streptococcus epidermidis*. Among the reported bio-active CDPs, cyclo(Pro-Phe), cyclo(Pro-Tyr), and their analogs had been identified as QS inhibitors from various microbial species. Chen and coworkers reported the isolation and identification of cyclo(Pro-Phe) from the fungus *Rosellinia necatrix* in 1960.^23^ José and co-workers found that *P. aeruginosa* can produce cyclo(*L*-Pro-*L*-Val), cyclo(*L*-Pro-*L*-Phe), and cyclo(*L*-Pro-*L*-Tyr), regulated by its *las*I QS system, thus promoting the plant growth.^24^ Abed and co-workers isolated cyclo(*L*-Pro-*L*-Phe) from *Marinobacter* sp. SK-3 of a hypersaline microbial mat with anti-QS activity inhibited QS-dependent production of violacein by *Chromobacterium violaceum* CV017, reduced QS-dependent luminescence of the reporter *Escherichia coli* pSB401 induced by 3-oxo-C6-HSL.^25^ In addition, the same cyclic dipeptide from *Bacillus amyloliquefaciens* Q-426 inhibited the formation of biofilms.^26^ Our group reported the isolation of cyclo(*L*-Pro-*L*-Phe) from *Sphingomonas* sp. WG,^27^ and cyclo(*L*-Pro-*L*-Tyr) from *Penicillium chrysogenum* DXY-1.^28^ We explored the anti-QS activity of the cyclo(*L*-Pro-*L*-Tyr) and its isomers, and found that altering the stereochemistry of this CDP reduced its biological activity. Apart from these two cyclic dipeptides, cyclo(*L*-Hyp-*L*-Tyr), which contains hydroxyproline, also attracted our interest. Cyclo(*L*-Hyp-*L*-Tyr) has already been reported as a natural product isolated from some microorganisms,^29^ with less study about its bioactivity. Structurally, the backbone of these three cyclic dipeptides is very similar, differing in the presence or absence of hydroxyl group at position *4′* of the tyrosine residue or at position *4* of the proline residue (Figure 1A). Compared with the linear form, CDPs generally show better biological activity because of their higher stability, protease resistance, and conformational rigidity. On the other hand, CDPs containing aromatic amino acids have also been reported to self-assemble into different morphologies.^30^^,^^31^

In this work, we compared the anti-QS activity of three synthetic CDPs cyclo(*L*-Pro-*L*-Tyr), cyclo(*L*-Hyp-*L*-Tyr), and cyclo(*L*-Pro-*L*-Phe) against *P. aeruginosa* PAO1, including the inhibition of virulence factors, swarming motility, and biofilm formation, as well as the effect on the expression of QS-related genes. Then, the binding affinity of these cyclic dipeptides to QS receptor protein LasR was investigated by molecular docking and Isothermal Titration Calorimetry (ITC) assay. In addition, cyclic dipeptides which contain aromatic moieties have been discovered displaying self-assembly behavior.^32^^,^^33^^,^^34^^,^^35^^,^^36^ However, the proline-containing sequences were not always easily aggregated, because of their more rigid structure. A series of cyclo(Phe-Xaa) has been reported forming hydrogel in an aqueous solution, except cyclo(Phe-Pro).^37^ We also wondered whether the introduction of hydroxyl group could affect the assemble ability and then further impact the anti-QS activity.

## Results and discussion

### The antimicrobial activity and cytotoxicity of CDPs

The three CDPs cyclo(*L*-Pro-*L*-Tyr), cyclo(*L*-Hyp-*L*-Tyr), and cyclo(*L*-Pro-*L*-Phe) were synthesized by GL Biochem (shanghai) Ltd and Anhui Guoping Pharmaceutical Co*.* Their structures and purity were confirmed by NMR and HPLC, and they were all purified using pre-HPLC before biological experiments.

To investigate the biocompatibility of CDPs to *P. aeruginosa*, the growth curve of PAO1 treated with these CDPs at concentrations of 1.8 mM, and 10 mM were investigated. As shown in Figure 1B, there was no significant difference in bacterial growth between the cyclic dipeptides treated samples and the blank control. The population of bacterial cells reached the logarithmic growth phase after about 4 h and reached the stable stage after 20 h. The results demonstrated that cyclic dipeptides had little influence on PAO1 growth at the experimental concentrations.

To further investigate the cytotoxicity of CDPs to mammalian cells. The cytotoxicity of cyclo(*L*-Pro-*L*-Tyr), cyclo(*L*-Hyp-*L*-Tyr), and cyclo(*L*-Pro-*L*-Phe) was evaluated on lung cancer A549 and mouse embryo NIH-3T3 using the MTT assay. At concentrations of less than 10 mM, all three cyclic dipeptides showed low toxicity to mammalian cells. At the same time, cyclo(*L*-Pro-*L*-Phe) had more toxicity to the cells than the other two cyclic dipeptides, which contain the hydroxyl group (Figures 1C and 1D).

### The effect on virulence factors production of *P. aeruginosa* PAO1

The QS system in *P. aeruginosa* regulates the production of virulence factors such as pyocyanin, elastase, proteases, and pyoverdin, as well as biofilm formation.^38^^,^^39^ To verify the anti-QS activity of these three CDPs against PAO1, we measured the production of the above virulence factors.

**Figure 2:**
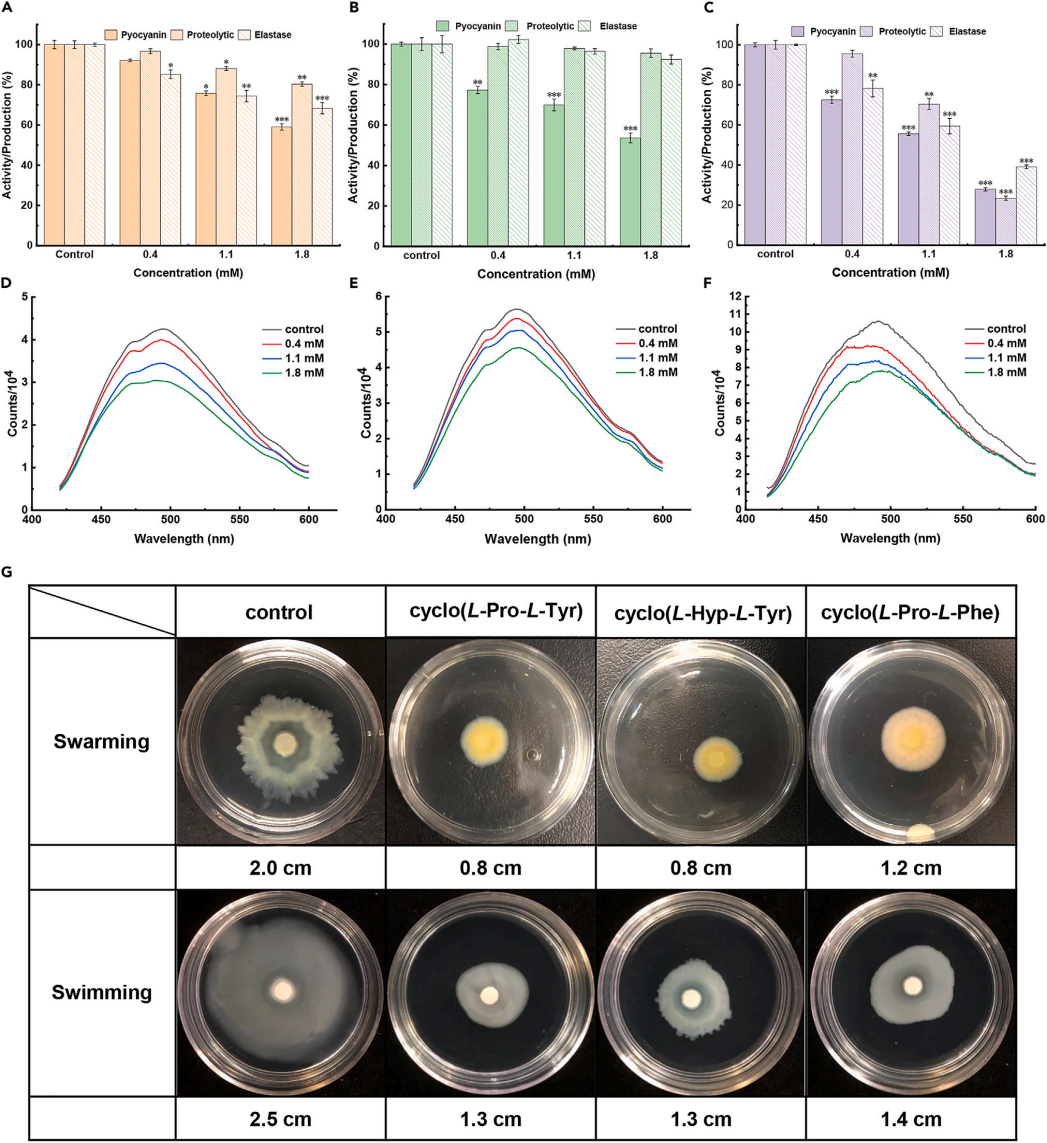
Anti-QS activities of CDPs against *P. aeruginosa* PAO1 Effect of cyclo(*L*-Pro-*L*-Tyr) (A and D), cyclo(*L*-Hyp-*L*-Tyr) (B and E), and cyclo(*L*-Pro-*L*-Phe) (C and F) on the production of virulence factors of *P. aeruginosa* PAO1 at different concentrations. Swarming and swimming motility (G) of *P. aeruginosa* PAO1 under treatment with PBS, 1.8 mM cyclo(*L*-Pro-*L*-Tyr), cyclo(*L*-Hyp-*L*-Tyr), and cyclo(*L*-Pro-*L*-Phe). Values are presented as mean ± SD (n = 3). The statistical significance was determined by using a one-way ANOVA test. ∗p < 0.05, ∗∗p < 0.005, ∗∗∗p < 0.0005 (versus control).

Based on our previous results,^28^ we chose the experimental concentrations of 0.4 mM, 1.1 mM, and 1.8 mM. As shown in Figures 2A–2C, the production of typical virulence factors decreased after the treatment with these three CDPs in a dose-dependent manner. At a concentration of 1.8 mM, cyclo(*L*-Pro-*L*-Tyr) decreased the pyocyanin production by 41%, and activities of proteases and elastase by 20% and 32%, respectively. For the treatment with cyclo(*L*-Hyp-*L*-Tyr), which consists of an additional hydroxyl group compared with cyclo(*L*-Pro-*L*-Tyr), the pyocyanin production decreased by 47%. In comparison, the activities of proteases and elastase were only inhibited by 5% and 8%. Cyclo(*L*-Pro-*L*-Phe), which lacks hydroxyl group, showed the best inhibitory efficiency on all these three virulence factors. After being treated with it, the production of pyocyanin, and activities of proteases and elastase were inhibited by 73%, 77%, and 61%, respectively. The results indicated that presence of hydroxyl groups inversely correlates with activity. Another important hallmark of *P. aeruginosa* virulence is the ability to produce low-molecular-weight iron-chelating compounds called pyoverdin, which aid in bacterial growth and survival under iron-limiting conditions. Changes in pyoverdin amount could be determined via measuring fluorescence intensity changes. The fluorescence intensity of the peptide-treated bacterial supernatant decreased in a dose-dependent manner at the experimental concentrations (Figures 2D–2F).

Many bacteria swim in liquid or swarm over solid surfaces by rotary flagella, wherein the inhibition of motility promotes biofilm formation.^40^ Then, the cyclic dipeptides were tested for their inhibition on the swarming and swimming of *P. aeruginosa* PAO1. As shown in Figure 2G, after being treated with 1.8 mM cyclo(*L*-Pro-*L*-Tyr), cyclo(*L*-Hyp-*L*-Tyr), and cyclo(*L*-Pro-*L*-Phe), both the swarming and swimming motility migration diameters were smaller than that of the control group. All three cyclic dipeptides seemed to inhibit the bacterial motility of PAO1, with no significant differences in inhibitory efficiency.

### The effects on biofilm formation

**Figure 3:**
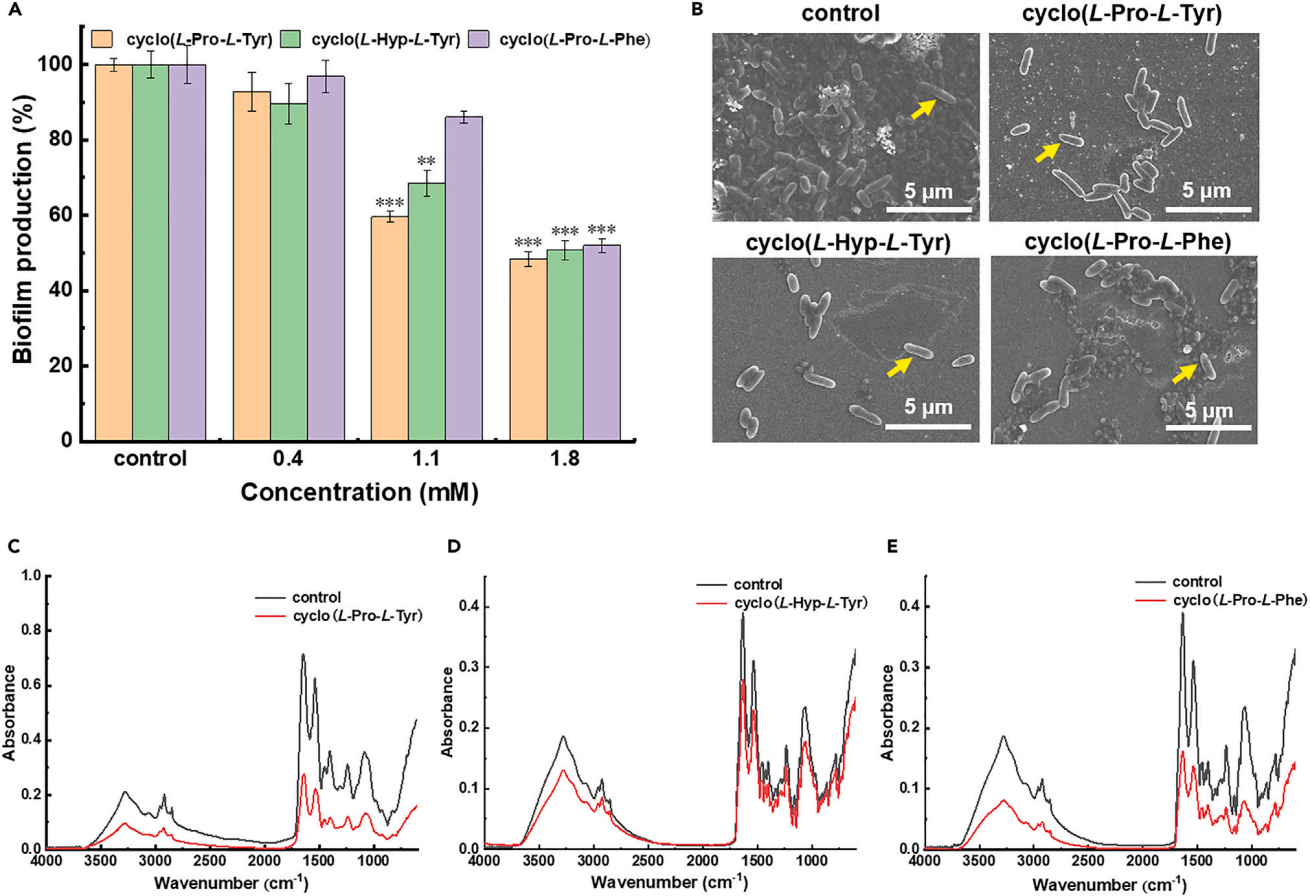
Anti-biofilm activity of CDPs on PAO1 (A) Biofilm formation of PAO1 under treatment with cyclo(*L*-Pro-*L*-Tyr), cyclo(*L*-Hyp-*L*-Tyr), and cyclo(*L*-Pro-*L*-Phe) at different concentrations. (B) SEM images of remaining PAO1 cells after treatment with cyclo(*L*-Pro-*L*-Tyr), cyclo(*L*-Hyp-*L*-Tyr), and cyclo(*L*-Pro-*L*-Phe) (1.8 mM). The intact short rods of PAO1 with clear inner and outer membranes are marked by yellow arrows. (C–E) ATR-FTIR spectra of bacterial cells' contents. Values are presented as mean ± SD (n = 3). PBS served as the blank control. The statistical significance was determined by using a one-way ANOVA test. ∗p < 0.05, ∗∗p < 0.005, ∗∗∗p < 0.0005 (versus control).

Bacterial biofilms are refuges for bacteria that significantly enhance their resistance to antibiotics and host immune defense mechanisms, leading to persistent and recurrent infections.^41^ The formation of dense biofilm is one of the leading causes of *P. aeruginosa* evolving drug resistance. The mechanism of biofilm formation is complex and is affected by many factors. It has been reported that in many bacteria, QS system can regulate bacterial biofilm formation.^42^^,^^43^ After being co-cultured with the three CDPs, the bacterial biofilm mass was measured using a crystal violet staining assay. As shown in Figure 3A, all the CDPs inhibited the biofilm formation at experimental concentrations in a dose-dependent manner. At the concentration of 1.8 mM, cyclo(*L*-Pro-*L*-Tyr), cyclo(*L*-Hyp-*L*-Tyr), and cyclo(*L*-Pro-*L*-Phe) inhibited biofilm formation by 52%, 50%, and 48%, respectively. Of interest, at the concentration of 1.1 mM, the difference in the biofilm inhibition ratio of these three cyclic dipeptides was more significant, in which cyclo(*L*-Pro-*L*-Phe) showed a weak inhibiting ability.

To observe the effect of cyclic dipeptides on bacterial morphology, the morphology of bacteria and biofilms was observed by scanning electron microscopy (SEM). For the PAO1 that was untreated with any CDPs, a thick biofilm could be observed covering the intact rod-shaped bacterial cell. Although for the samples being treated with CDPs, the biofilm was destroyed, and the bacteria looked planktonic. Notably, the bacterial cells also showed normal morphology with clear and complete inner and outer membranes and thin and uniform periplasmic space entirely in the form of short rods, which indicated that these CDPs only inhibit the biofilm formation without damaging the bacterial cells.

To further investigate the reduction in bacterial biofilms, we performed ATR-FTIR analysis on changes in the bacterial cellular components after CDPs treatment.^27^^,^^44^ There are four major typical absorbance prominent regions representing different components of the biofilm (3700-3100 cm^−1^ corresponding to the hydration, 3050-2800 cm^−1^ to fatty acids, 1750-1500 cm^−1^ to amide linkages within proteins and peptides, 1500-1000 cm^−1^ to a mixed region of proteins, fatty acids and nucleic acids, respectively).^45^^,^^46^^,^^47^ As shown in Figures 3C–3E, compared with the control group, CDPs-treated PAO1 cells showed prominently declined absorbance in the four major regions, indicating that the carbohydrate, fatty acid, protein, and nucleic acids contents of the bacterium were reduced. Also, the bacterial-secreted cellular components were relatively reduced, leading to a decrease in bacterial adhesion. This indirectly indicated that the CDPs had some inhibitory activity on biofilm formation of PAO1.

### The effects on QS-regulated genes of PAO1

**Figure 4:**
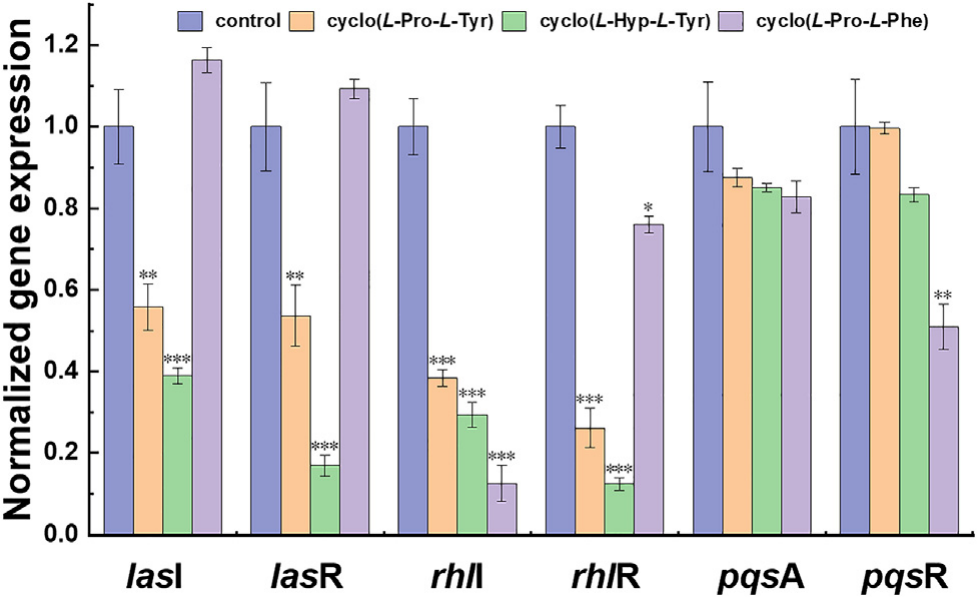
The effects of CDPs on PAO1 QS genes expression The *rpo*D was used as an internal standard. PBS served as the control. Values are presented as mean ± SD (n = 3). Statistical analysis was performed using a one-way ANOVA test. ∗p < 0.05, ∗∗p < 0.005, ∗∗∗p < 0.0005 (versus control).

It has been documented that three main pathways participated in *P. aeruginosa* QS system. The *las* and *rhl* systems mainly control the production of pyocyanin, the expression of elastase and proteases, and the maturation stage of biofilm formation; the *pqs* system is mainly involved in the early biofilm formation.^16^ RT-PCR was then employed to evaluate specifically to assess the impacts on the QS genes’ expression, investigating the possible anti-QS mechanism of these CDPs. As shown in Figure 4A, cyclo(*L*-Pro-*L*-Tyr) and cyclo(*L*-Hyp-*L*-Tyr) exhibited similar regulating predictions of the test genes, and cyclo(*L*-Hyp-*L*-Tyr) showed a better down-regulating capacity of genes expression of *las* and *rhl* system, whereas these two CDPs displayed less effect on the *pqs* system. The cyclo(*L*-Pro-*L*-Phe), which substitutes the hydroxyl group for hydrogen, mainly influenced the expression of *rhl*I, and had some effect on *pqs*R. The results indicated cyclo(*L*-Pro-*L*-Tyr) and cyclo(*L*-Hyp-*L*-Tyr) could inhibit biofilm formation and reduce virulence factor production by affecting both *las* and *rhl* systems. In comparison, cyclo(*L*-Pro-*L*-Phe) acted mainly by targeting the *rhl* system.

### Binding analysis for LasR and CDPs

**Figure 5:**
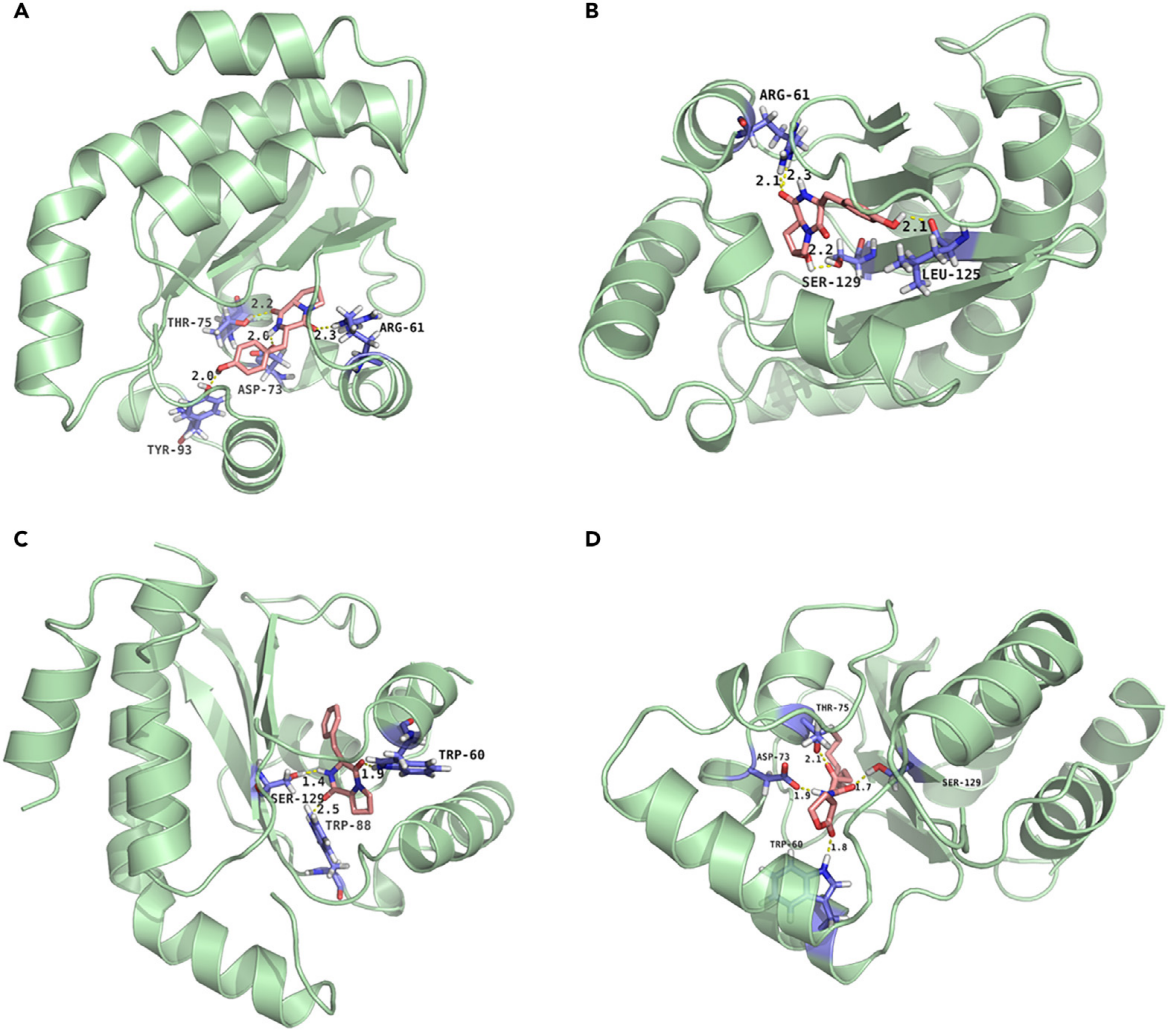
Molecular docking of CDPs to LasR Docked conformation of cyclo(*L*-Pro-*L*-Tyr) (A), cyclo(*L*-Hyp-*L*-Tyr) (B), cyclo(*L*-Pro-*L*-Phe) (C), and natural ligand 3OC12-HSL (D) with LasR (2UV0) receptor proteins respectively. The hydrogen bonds formed were presented as yellow dashed lines.

To study the possible inhibitory mechanism of CDPs on the QS system of *P. aeruginosa* PAO1, molecular docking was performed to explore the interaction of the three cyclic dipeptides with the QS receptor LasR. Docking stimulation was performed using LasR (PDB: 2UV0) (Figure 5). The docking between natural ligand N-3-oxo-dodecanoyl homoserine lactone (3OC12-HSL) and LasR was regarded as a control, and the docking box was set containing the whole protein simultaneously. The docking result of 3OC12-HSL was highly consistent with the reported X-ray structures (Figure 5D)^48^^,^^49^ and previous study,^50^ demonstrating the construction of a reliable docking method. The docking data indicated that these three CDPs could bind with LasR in the same region as the ligand. All the binding energy of three cyclic dipeptides cyclo(*L*-Pro-*L*-Phe) (−7.99 kcal/mol), cyclo(*L*-Hyp-*L*-Tyr) (−8.13 kcal/mol), cyclo(*L*-Pro-*L*-Tyr) (−8.28 kcal/mol), were slightly higher than that of the natural ligand (−8.33 kcal/mol). The amino acids of LasR surrounding the small molecules which form hydrogen bonding were listed in Table 1.

**Table 1:** Detailed docking information of LasR with different CDPs and autoinducer 3OC12-HSL

Molecules	Binding energy (kcal/mol)	Hydrogen binding interactions
cyclo(*L*-Pro-*L*-Tyr)	−8.28	Arg61, Asp73, Thr75, Tyr93
cyclo(*L*-Hyp-*L*-Tyr)	−8.13	Arg61, Leu125, Ser129
cyclo(*L*-Pro-*L*-Phe)	−7.99	Trp60, Trp88, Ser129
3OC12-HSL	−8.33	Trp60, Asp73, Thr75, Ser129

**Figure 6:**
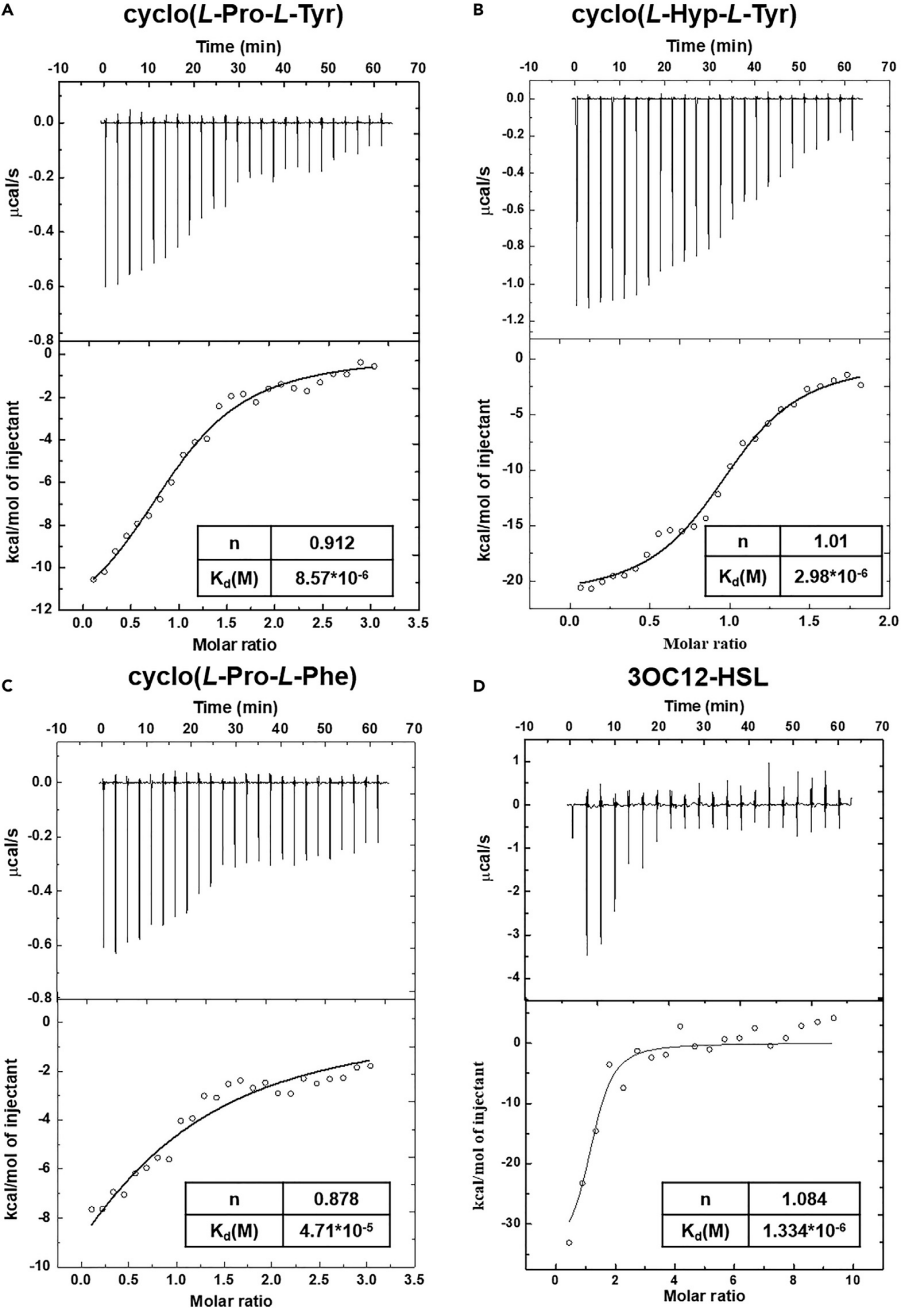
Microcalorimetric binding studies of LasR with CDPs (A–C) Titration of 8 μM LasR with 200 μM CDPs. (D) Titration of 8 μM LasR with 200 μM 3OC12-HSL. The upper panel shows the raw titration data. The lower panel shows dilution-heat corrected and concentration-normalized integrated peak areas of the raw titration data. Data were fitted with the “Independent” model of the Launch ITC Run software.

Next, the interaction of these three CDPs with LasR protein was further verified by isothermal titration calorimetry (ITC). LasR was expressed and purified without the natural ligand. As shown in Figure 6, the binding constant (K_d_) of natural ligand 3OC12-HSL was 1.33 μM, which is consistent with previous reports.^50^^,^^51^ The binding number is about 1, suggesting that one protein monomer bind one ligand. The three cyclic dipeptides displayed the same binding stoichiometry with LasR. Cyclo(*L*-Hyp-*L*-Tyr) and cyclo(*L*-Pro-*L*-Tyr) also showed micromolar-range binding affinities for LasR. However, the binding affinity of cyclo(*L*-Pro-*L*-Phe) for LasR was much lower, which was consistent with the docking results. Combining the anti-QS activity and gene expression analysis, and the interaction data, we hypothesized that there might be other targets that cyclo(*L*-Pro-*L*-Phe) acted in the QS system.

### Self-assembly assay

**Figure 7:**
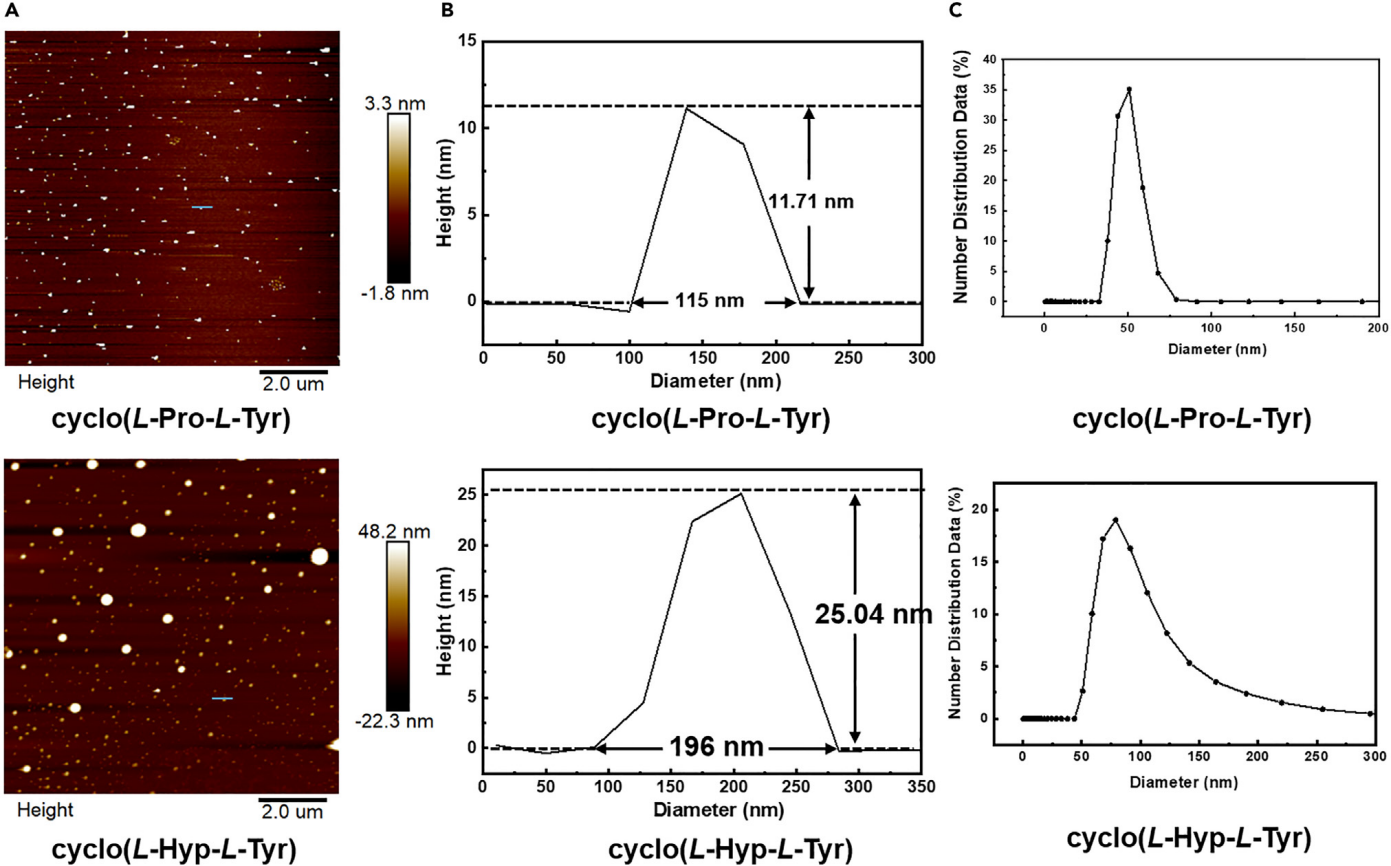
Self-assembly properties of CDPs AFM images of CDPs (A), the height of the aggregates along the blue lines in the AFM images (B) and DLS (C) of cyclo(*L*-Pro-*L*-Tyr), cyclo(*L*-Hyp-*L*-Tyr), and cyclo(*L*-Pro-*L*-Phe) at 1.8 mM.

Intrinsic fluorescence spectrometry and conductivity were used to measure the critical aggregation concentrations (CACs) of these three cyclic dipeptides. There was no change in the shape of the fluorescence emission spectra at the tested concentrations for all samples. The maximum emission wavelengths of cyclo(*L*-Pro-*L*-Tyr), cyclo(*L*-Hyp-*L*-Tyr), and cyclo(*L*-Pro-*L*-Phe) were located at ∼302, 303, and 283 nm, respectively. As the concentration of CDPs solution gradually increased, the fluorescence intensity first increased and then weakened (Figure S8A), and the turning points indicated the occurrence of aggregating. The CAC values of cyclic dipeptides inferred from the fluorescence intensity or conductivity changes of the peaks (Figures S8 and S9) are listed in Table 2. It was obvious that the hydroxyl group rendered cyclo(*L*-Pro-*L*-Tyr) and cyclo(*L*-Hyp-*L*-Tyr) more easily to aggregate. Subsequently, after incubation for 24 h, the morphology and size of the aggregates were measured by Atomic Force Microscope (AFM) and Dynamic Light Scattering (DLS). As shown in Figure 7, cyclo(*L*-Pro-*L*-Tyr) and cyclo(*L*-Hyp-*L*-Tyr) showed spherical morphology with a particle size of 50 nm and 100 nm, respectively. Cyclo(*L*-Pro-*L*-Phe) could not be detected obviously by AFM and the dimensions measured with DLS was extremely small, about 1∼2 nm. Apparently, the CAC values of cyclo(*L*-Pro-*L*-Tyr) and cyclo(*L*-Hyp-*L*-Tyr) were both less than the maximum tested concentration (1.8 mM), indicating that these two cyclic dipeptides functioned as aggregates in the bioactivity assys. In the contrast, the CAC value of cyclo(*L*-Pro-*L*-Phe) was much higher than the maximum tested concentration, indicating that it acted as a QS inhibitor as a monomer.

**Table 2:** Experimentally measured CACs of CDPs

Molecules	CACs (mM)	
	fluorescence spectra	conductivity
cyclo(*L*-Pro-*L*-Tyr)	1.1	0.9
cyclo(*L*-Hyp-*L*-Tyr)	0.8	0.5
cyclo(*L*-Pro-*L*-Phe)	5.4	4.1

It has been studied that, molecular self-assembly may affect its biological activity.^52^^,^^53^^,^^54^ Many cationic antimicrobial peptides always could self-assemble in aqueous solution, and thus increased their antimicrobial activity.^55^^,^^56^ The QS signals, acyl-homoserine lactones, were also proved to self-assemble into micelles and vesicles in aqueous solutions, and the aggregates were suggested could play roles in mediating these signals’ transportation between cells.^57^ We speculated that the differences in the anti-QS and anti-biofilm activities of these cyclic peptides could also be caused by molecular self-assembly.

### Limitations of the study

Here, we demonstrate the influence of the hydroxyl group on the bioactivity and self-assembly of three synthetic structure-similar cyclic dipeptides. Although the genes expression result proved that cyclo(*L*-Pro-*L*-Phe) acted as a QSI in a different way from the other two hydroxyl-containing cyclic dipeptides, the reason for the difference is still needed to explore in the future study. Moreove, detailed understandings on the action mechanisms of cyclic dipeptides as QSIs are still needed in the future.

## STAR★Methods

### Key resources table

**Table:** 

REAGENT or RESOURCE	SOURCE	IDENTIFIER
**Bacterial and virus strains**		

*P. aeruginosa* PAO1	ATCC	ATCC 27853
Experimentalmodels Cell lines		
A549	ATCC	CCL-185
NIH-3T3		

**Chemicals, peptides, and recombinant proteins**		

PBS(PBS)	Sigma-Aldrich	P5493
Dulbecco’s Modified Eagle Medium (DMEM)	Damas life	P1872015
RNAiso Plus	Takara	108-95-2
Pancreatin	Damas life	P1956729
3-(4,5-Dimethyl-2-Thiazolyl)-2,5-Diphenyl Tetrazolium Bromide (MTT)	Aladdin	298-93-1
Azocasein	Sigma-Aldrich	102110-74-7
Elastin-Congo red (ECR)	Bomei	AR
Tryptone	OXOID	
Yeast Extract	OXOID	
Crystal Violet	SINOPHARM	AR

**Critical commercial assays**		

iScriptTM cDNA Synthesis kit	TaKaRa	6210A
Corning-96 Cell culture plates	Corning	3365

**Software and algorithms**		

Auto Dock Tools-1.5.6	AutoDock Inc	http://mgltools.scripps.edu/
using OriginPrio 9.1 software	OriginLab	https://www.originlab.com/

### Resource availability

#### Lead contact

Further information and requests for resources and reagents should be directed to and will be fulfilled by the lead contact, Zhu. Hu (zhuhu@fjnu.edu.cn).

#### Materials availability

This study did not generate new unique reagents.

### Experimental model and subject details

#### Bacterial strain and culture conditions

*P. aeruginosa* PAO1 (ATCC 27853) strain was used to evaluate anti-QS activity. PAO1 was incubated in LB medium (tryptone 1% w/v, yeast extract 0.5% w/v, NaCl 1% w/v and agar 1% w/v) overnight at 37°C at 165 rpm.

#### Cell culture

Human lung epithelial cell line A549 (ATCC CCL-185) was a kind donation from Prof. Cuixia Chen of China University of Petroleum (East China). Mouse embryonic fibroblasts NIH-3T3 were donated by Dr. Hanping Fu from Fujian Normal University. A549 and NIH-3T3 cells were proliferated in F-12K or Dulbecco’s Modified Eagle’s Medium (DMEM) with 1% Penicillin-Streptomycin and 10% heat-inactivated fetal bovine serum at 37°C under 5% CO_2_.

### Method details

#### Growth curve of *P. aeruginosa* PAO1

The growth curve of PAO1 was measured using a microplate reader (BioTek, SynergyTM LX, USA) under treatment with different CDPs (1.8 mM or 10 mM). Briefly, the overnight cultures of PAO1 were diluted with LB medium to OD_600_ = 0.05, then inoculated in 96-well plate with CDPs at 37°C. PBS was used as a negative control. The bacterial density was measured every 2 h up to 24 h at a wavelength of 600 nm.

#### Cytotoxicity activity

The MTT method was used to perform a cytotoxicity experiment.^58^ A549 or NIH-3T3 cells (3000 cells per well) were seeded into a 96-well plate and incubated at 37 for 24 h under 5% CO_2_ atmosphere. The cells were then treated without and with the CDPs in different concentrations ranging from 0.5 to 10 mM and cultured for 24 h under the same cultivating conditions. After 24 h, the culture-attached cells were washed twice with the medium. One hundred microlitre of 3-(4,5-Dimethyl-2-Thiazolyl)-2,5-Diphenyl Tetrazolium Bromide (MTT) dissolved in the medium at a 1 mg/mL concentration was added to every well and co-cultured with the cells for further 4 h. Culture was removed, and DMSO was added to dissolve the generated formazan. The plates were incubated at 37°C for 20 min on a shaker at 120 rpm, and the absorbance (OD_570_) was determined by a microplate reader. The cytotoxicity of the CDPs was evaluated using the relative viability of the cell, for which the blank control value was 100%. Every tested concentration was repeated independently three times.

#### QS-regulated virulence factors assay

The virulence factors were determined according to the previous method^59^ with a slight modification. PAO1 suspensions (OD_600_ = 0.05) were incubated with sub-MIC doses (0.4 mM, 1.1 mM, and 1.8 mM) of CDPs in each 10 mL conical flask at 37°C at 180 rpm for 12 h. After incubation, the culture media was centrifuged at 12000 rpm for 10 min to remove the cell precipitation.

##### Pyocyanin assay

The 5 mL supernatant was extracted with 3 mL CHCl_3_, and the organic layer was then extracted with 1 mL HCl (0.2 M). The upper water phase was collected via centrifugation, and the absorbance was measured at 520 nm. The effects of the compounds were evaluated using the relative pyocyanin production, for which the blank control value was 100%.

##### Proteolytic activity assay

The supernatant (100 μL) was reacted with 0.8% azocasein in a buffer (400 μL 50 mM K_2_HPO_4_, pH 7.0), and incubated at 30°C for 3 h. Afterward, 1.5 M HCl was added to the mixture and reacted on ice for 10 min. The solution was centrifugated at 12000 rpm for 10 min and the OD_440_ of the supernatant was determined. The effects of the compounds were evaluated using the relative production of degraded azocasein, for which the blank control value was 100%.

##### Elastase activity assay

The 100 μL supernatant was shaken at 37°C for 12 h with 5 mg Elastin-Congo red (ECR) and 400 μL Na_2_HPO_4_ (pH 7.2). After that, the reaction mixture was centrifuged at 12000 rpm for 10 min to obtain a clear supernatant, and the OD_495_ was determined. The effects of the compounds were evaluated using the relative production of degraded ECR, for which the blank control value was 100%.

##### Pyoverdin activity assay

Fluorescence intensity in the supernatant was measured using a fluorescence spectrophotometer at 405 nm excitation wavelength and 465 nm emission wavelength.

#### Swarming and swimming motility assay

*P. aeruginosa* PAO1 was investigated using the following media: (1) swarming plate (0.5% w/v tryptone, 0.2% w/v yeast extract, 1% w/v glucose, 0.5% w/v agar), (2) swimming plate (1% w/v tryptone, 0.5% w/v NaCl, 0.3% w/v agar). The agar media was cooled down for 10 min before use and different concentration CDPs were added. After solidification, a sterile filter paper with a diameter 2 mm was placed in the center with 2 μL overnight culture of PAO1 suspensions (1 × 10^7^ CFU mL^−1^), and incubated at 37°C for 24 h. The colony phenotype was observed and the colonies were imaged.

#### Biofilm assay

The effect of CDPs on biofilm formation was evaluated by the crystal violet staining method following bacterial growth for 24 h in culture-treated 96-well flat-bottom plates as previously described.^60^ Briefly, bacterial cell suspensions (OD_600_ = 0.05) were incubated at 37°C with or without CDPs to allow biofilm formation. This assay was repeated three times. After the culture microplates recorded the optical density of 600 nm to monitor microbial growth, the wells of the contents removed were washed three times with 250 μL of sterile phosphate buffer saline (PBS) solution and stained with 200 μL crystal violet (0.1%) aqueous solution for 20 min. The excess stain was removed by washing with PBS and the plate was then dried at ambient temperature in air. A 200 μL ethanol (75%) was added to each well to redissolve the dye, and the biofilm was quantified by measuring the absorbance at 595 nm.

#### SEM

The visualization biofilms were observed under SEM to further confirm the effect of CDPs. Sterile cover glasses were placed in the bottom of 6- well plate, and then 2 mL *P. aeruginosa* PAO1 bacterial suspension (OD_600_ = 0.05) was cultivated at 37°C for 48 h. The coverslips were washed with PBS to remove the non-adherent cells, cultured in 2.5% glutaraldehyde for 4 h, and continuously dehydrated for 10 min with gradient ethanol series (50–90%). Following that, gold was sputtered on the dry biofilm’s cover for scanning electron microscopy.

#### ATR-Fourier transform infrared spectroscopic (FTIR) analysis

The bacterial suspension of *P. aeruginosa* PAO1 (OD_600_ = 0.05) was grown in a 6-well plate with or without an appropriate dose of CDPs, and the plate was subsequently cultured at 37°C for 2 days. The collected bacterial cells were freeze-dried after incubation. An ATR-FTIR spectrophotometer (Nicolet iS5, Thermo Scientific, USA) was used to test the ATR-FTIR spectra.

#### Expression of QS genes

Influence upon the expression levels of QS regulatory genes *las*I, *las*R, *rhl*I, *rhl*R, *pqs*A, and *pqs*R in *P. aeruginosa* PAO1 was determined by performing quantitative real-time PCR. *P. aeruginosa* PAO1 suspensions (OD_600_ = 0.05) were incubated with different concentrations of CDPs in 10 mL of LB liquid medium, before the RNA extraction was carried out according to the instructions of the RNAiso Plus (Total RNA extraction reagent) kit provided by TaKaRa Company. The total RNA was reverse recorded regarding the iScriptTM cDNA synthesis kit. Fluorescence qRT-PCR was performed with the iTaqTM Universal SYBR Green Supermix kit using a CFX96 Real-Time system. The following program was used for these genes: 95°C for 3 min followed by 40 cycles of 95°C for 30 s, 60°C for 30 s. The dissolution curve and amplification curve were observed. The experiment was repeated 3 times, the average value was taken, and the data were analyzed by relative quantitative 2^−ΔΔCt^ method with *rpo*D ribosomal gene (an internal control). The sequence detail illustrated in Table S1.

#### Isothermal titration calorimetry (ITC) assay

LasR expression and purification were performed as previously described.^51^ CDPs (200 μM) in the TSB buffer were titrated into LasR protein (8 μM) dissolved in the same buffer using a Nano ITC (TA, America) instrument at 25°C. The reference parameter was set to 22 injections (2 μL per injection) with a spacing of 200 s between each injection. Each experiment's first injection (0.4 μL) was discarded, and the collected data were analyzed with the Launch ITC Run software.

#### Critical aggregation concentration (CAC) measurements

In this experiment, the intrinsic fluorescence signal of cyclic dipeptides was used to determine its fluorescence spectrum and critical aggregation concentration (CAC). CDPs (4 mL) solutions with concentrations of 0.01 mM, 0.1 mM, 0.25 mM, 0.5 mM, 1 mM, 1.5 mM, 2 mM, 2.5 mM, 5 mM, 10 mM were made directly in aqueous solution. The above concentrations of cyclic dipeptide solutions were vortexed for 30 s to mix well, and the samples were worth to be tested after 12–24 h at room temperature and protected from light. The parameters of the fluorescence spectrum were set as follows: the excitation wavelength of cyclo(*L*-Pro-*L*-Tyr) was 274 nm, and the recorded wavelength range was 284–400 nm. The excitation wavelength of cyclo(*L*-Hyp-*L*-Tyr) was 274 nm, and the recorded wavelength range was 295–400 nm. The excitation wavelength of cyclo(*L*-Pro-*L*-Phe) was 257 nm, the recorded wavelength range was 270–350 nm. The final CAC values were calculated by plotting the fluorescence intensity at the maximum emission wavelength or conductivity as the vertical coordinate and logc as the horizontal coordinate.

#### Atomic Force Microscope (AFM)

AFM was used to examine the morphology of CDPs’ assemblies. CDPS solutions were configured at ambient temperature and dropped (10 μL) onto mica sheets and dried at room temperature. Observations were carried out on a Bruker MultiMode 8 high-resolution AFM, where the morphology was characterized in ScanAsyst tip measurement mode at an elastic constant of 0.4 N/m for the silicon probe and a resonance frequency of 70 kHz.

#### Dynamic Light Scattering

The particle size of the 1.8 mM CDP_S_ aqueous solution was studied using Malvern Nano ZSE nanoparticles (Malvern Instruments Ltd., Worcestershire, UK) analyser. Test conditions: at a fixed scattering angle of 107° and a fixed temperature of 25°C, the scans were repeated three times with a cumulative total of 100 scans each. The data were processed by cumulative analysis of the experimental correlation function and the particle diameters were calculated from the calculated diffusion coefficients. Each reported measurement was performed three times.

### Quantification and statistical analysis

We determined differences in data between groups by one-way ANOVA. Comparisons of means between multiple groups were assessed by a one-way ANOVA test. All statistical analyses were carried out using Origin software version 9.1. Details and types of statistical analyses can be found in the figure legends. In this study, the statistical significance was determined by using a one-way ANOVA test. ∗p < 0.05, ∗∗p < 0.005, ∗∗∗p < 0.0005 (vs control).

## Data Availability

• All data produced in this study are included in the published article and its supplemental information, or are available from the lead contact upon request. • This paper does not report original code. • Any additional information required to reanalyze the data reported in this paper is available from the lead contact upon request.
