# Supplementary material for: Effects of hydroxyl group in cyclo(Pro-Tyr)-like cyclic dipeptides on their anti-QS activity and self-assembly
Source: iScience. 2023 Jun 7;26(7):107048. doi: 10.1016/j.isci.2023.107048 (PMC10285644; doi:10.1016/j.isci.2023.107048)
Supplement: Document S1. Figures S1–S9 and Table S1 [file mmc1.pdf]

iScience, Volume *26*

Supplemental information

Effects of hydroxyl group in cyclo(Pro-Tyr)-like cyclic dipeptides on their anti-QS activity and self-assembly

Li Li, Zuxian Xu, Ruipin Cao, Jiaxin Li, Chang-Jer Wu, Yinglu Wang, and Hu Zhu

SUPPLEMENTARY FIGURES

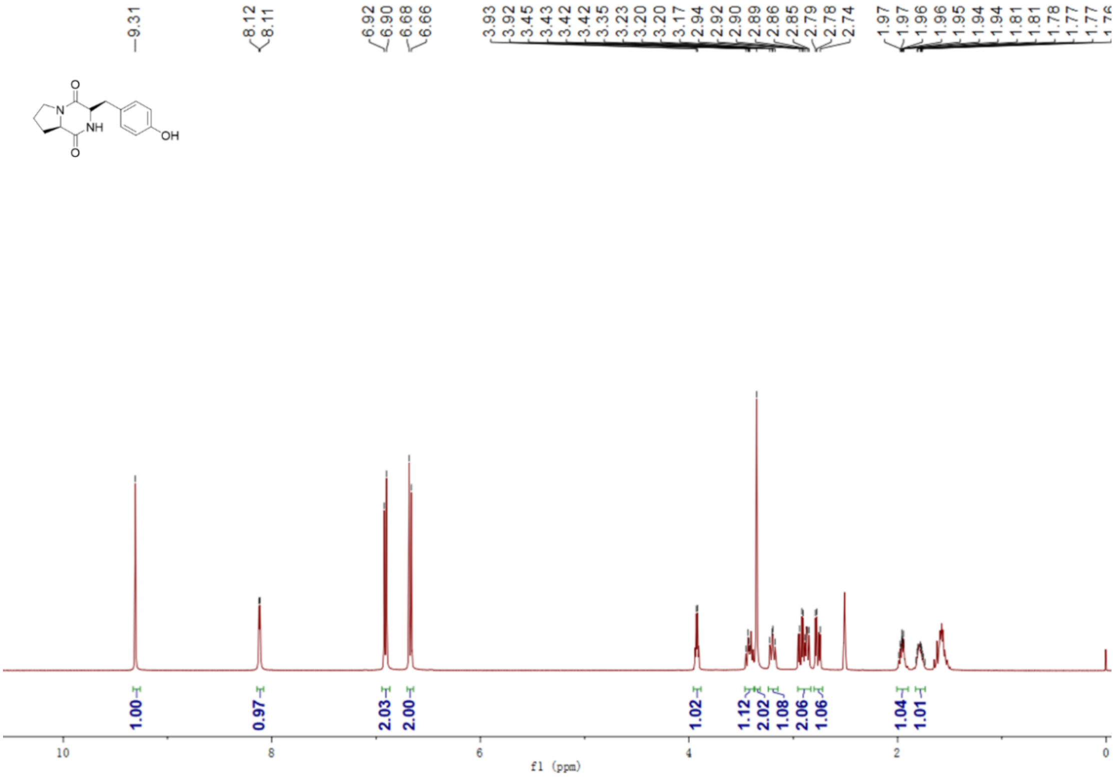

Figure S1. cyclo(*L*-Pro-*L*-Tyr) - ^{1}H NMR Spectrum - DMSO-d_6, 400 MHz, related to Figure 1.

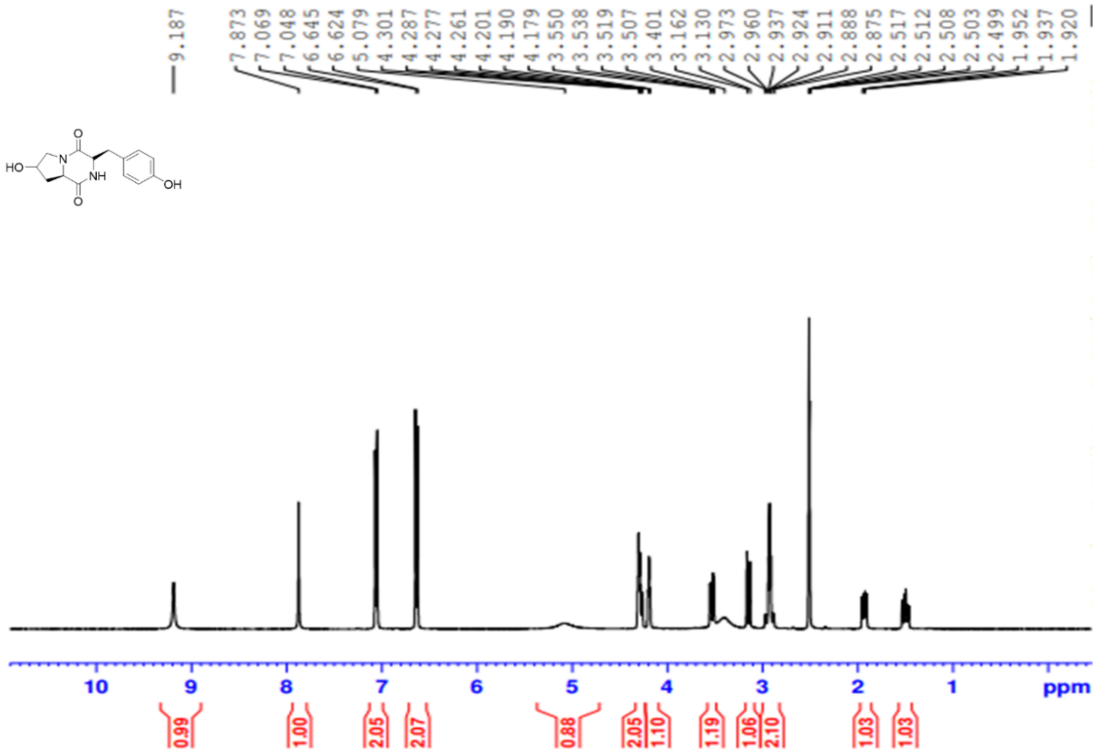

Figure S2. cyclo(*L*-Hyp-*L*-Tyr) - ^{1}H NMR Spectrum - DMSO-d_6, 400 MHz, related to Figure 1.

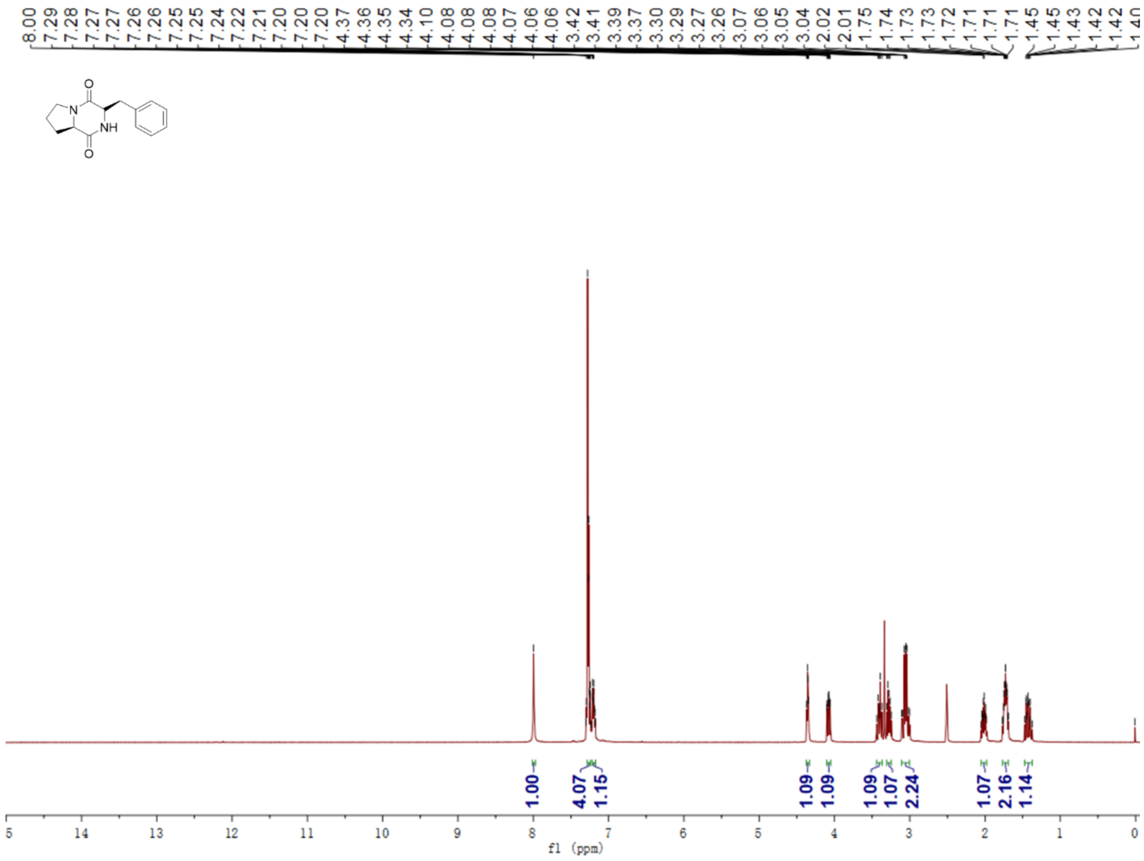

Figure S3. cyclo(*L*-Pro-*L*-Phe) - ^{1}H NMR Spectrum - DMSO-d_6, 400 MHz, related to Figure 1.

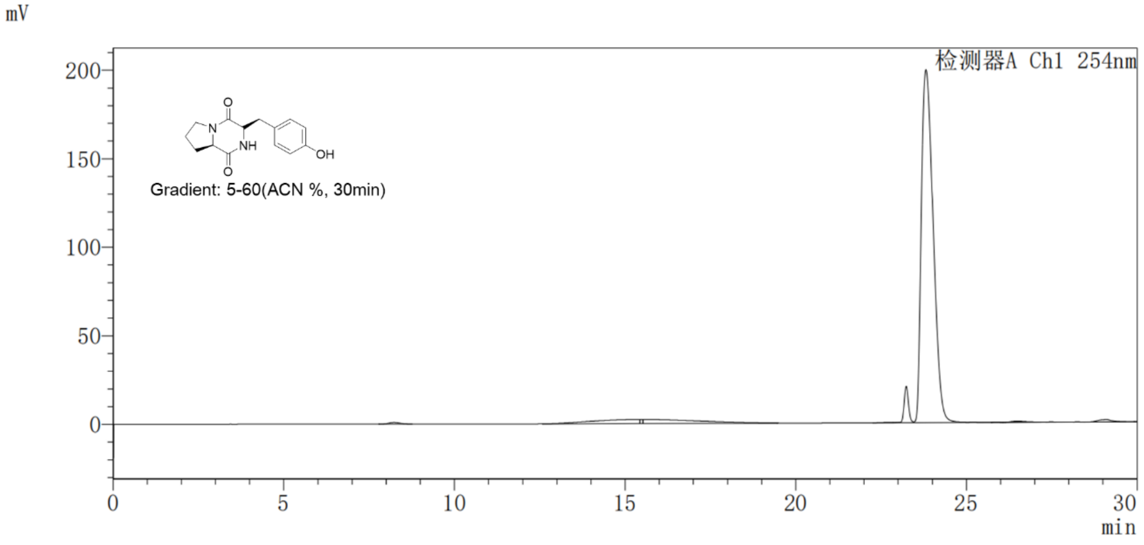

Figure S4. HPLC analsis of cyclo(*L*-Pro-*L*-Tyr), related to Figure 1.

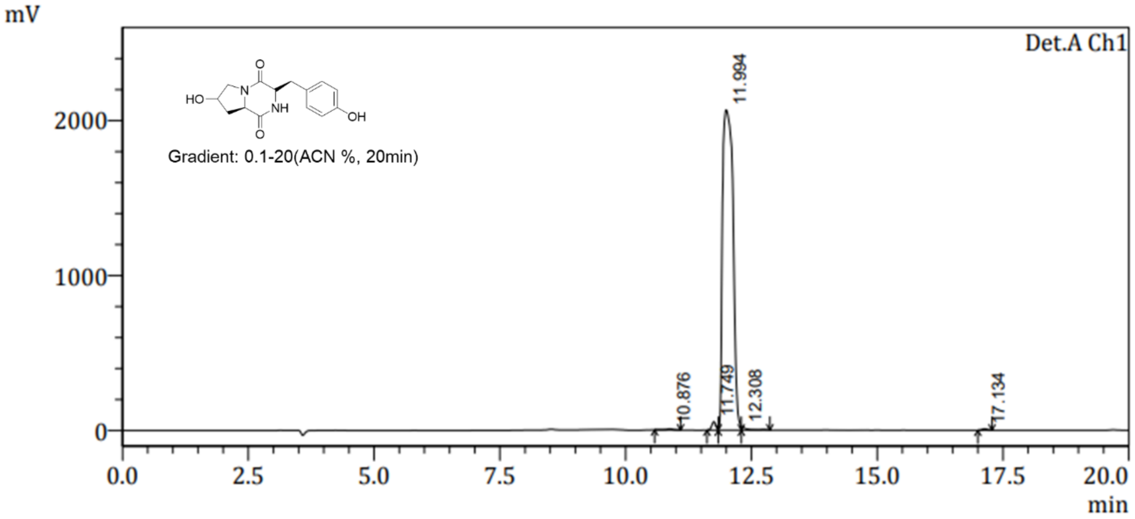

Figure S5. HPLC analsis of cyclo(*L*-Hyp-*L*-Tyr), related to Figure 1.

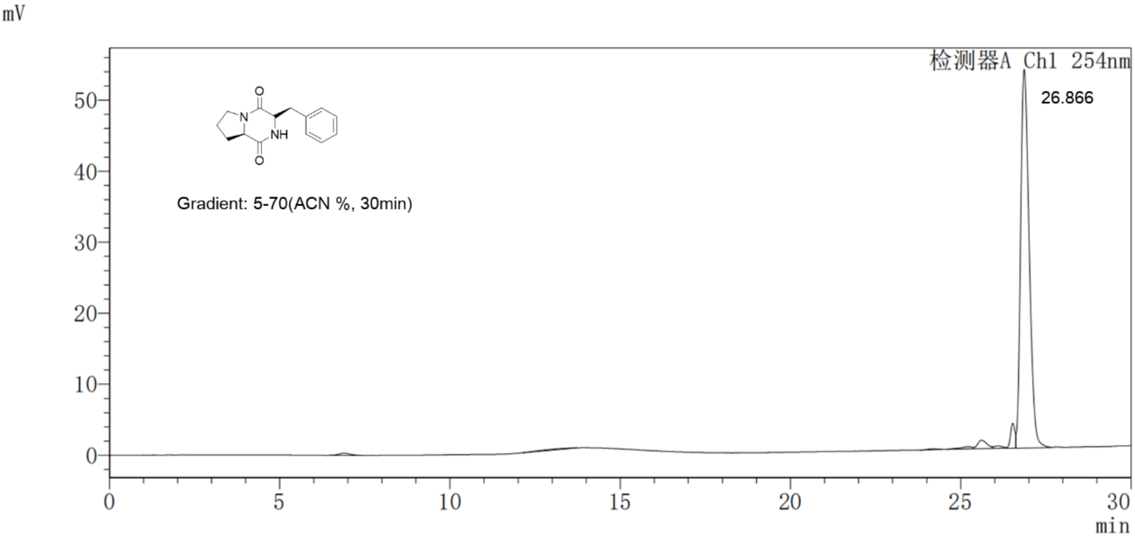

Figure S6. HPLC analsis of cyclo(*L*-Pro-*L*-Phe), related to Figure 1.

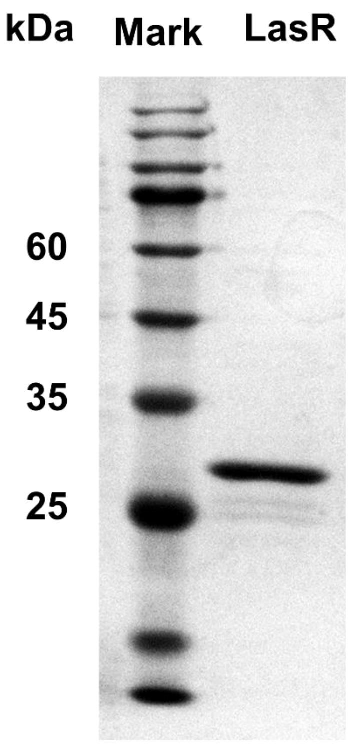

Figure S7. Sodium dodecyl sulfate-polyacrylamide gel electrophoresis (SDS-PAGE) analysis of purified LasR, related to Figure 6.

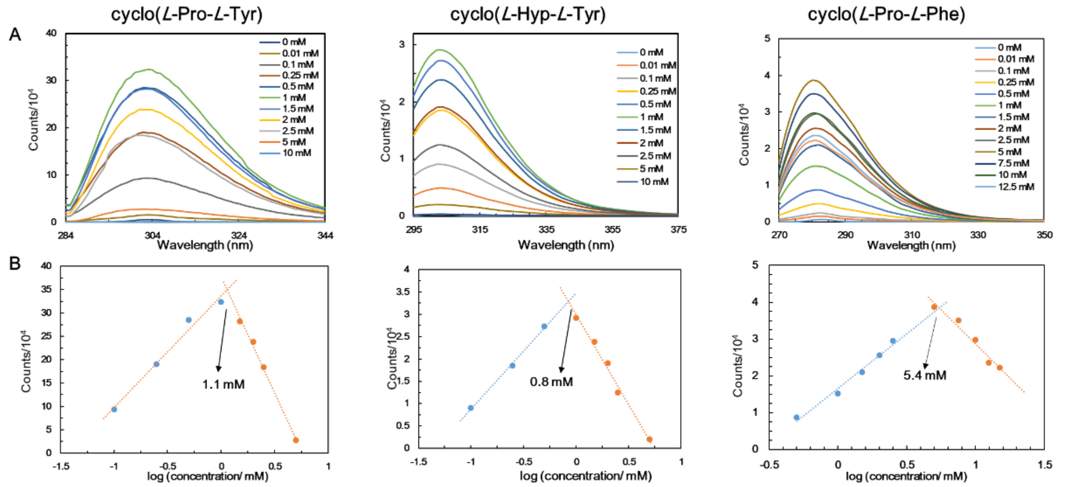

Figure S8. (a) The fluorescence spectra of different concentrations of CDPs at their respective excitation wavelengths. (b) The CAC was calculated using logc as the horizontal coordinate and the fluorescence intensity of the maximum emission wavelength as the vertical scale, related to Table 2.

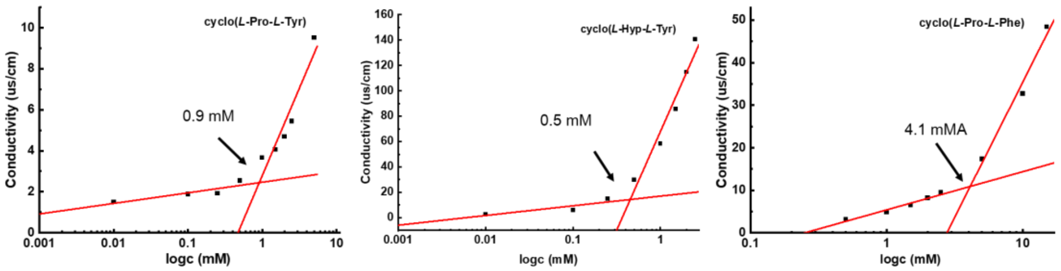

Figure S9. CAC of CDPs by conductivity testing, related to Table 2.

SUPPLEMENTARY TABLE

Table S1. Specific amplification primer sets for *P. aeruginosa* isolates, related to Figure 4.

Gene name	Type of primer	Primer Sequence (5'-3)
*las*I	Fw	CGTGCTCAAGTGTTCAAGGA
	Rev	GCGTCTGGATGTCGTTCTG
*las*R	Fw	CTGTGGATGCTCAAGGACTAC
	Rev	ACCGAACTTCCGCCGATT
*rhl*I	Fw	GCTACATCGTCGCCATGAG
	Rev	TCTCGCCCTTGACCTTCTG
*rhl*R	Fw	CCGATGCTGATGTCCAACC
